# Three Vessel Coronary Cameral Fistulae Associated with New Onset Atrial Fibrillation and Angina Pectoris

**DOI:** 10.1155/2014/475325

**Published:** 2014-02-19

**Authors:** Murat Yuksel, Abdulkadir Yildiz, Mustafa Oylumlu, Nihat Polat, Halit Acet, Necdet Ozaydogdu

**Affiliations:** ^1^Department of Cardiology, Faculty of Medicine, Dicle University, Diyarbakir, Turkey; ^2^Dicle Üniversitesi, Kalp Hastanesi 1.Kat, Kardiyoloji Kliniği, Sur, 21280 Diyarbakir, Turkey

## Abstract

Coronary cameral fistulas are abnormal communications between a coronary artery and a heart chamber or a great vessel which are reported in less than 0.1% of patients undergoing diagnostic coronary angiography. All three major coronary arteries are even less frequently involved in fistula formation as it is the case in our patient. A 68-year-old woman was admitted to cardiology clinic with complaints of exertional dyspnea and angina for two years and a new onset palpitation. Standard 12-lead electrocardiogram revealed atrial fibrillation (AF) with a ventricular rate of 114 beat/minute and accompanying T wave abnormalities and minimal ST-depression on lateral derivations. Transthoracic echocardiographic examination was normal except for diastolic dysfunction, minimally mitral regurgitation, and mild to moderate enlargement of the left atrium. Sinus rhythm was achieved by medical cardioversion with amiodarone infusion. Coronary angiography revealed diffuse and multiple coronary-left ventricle fistulas originating from the distal segments of both left and right coronary arterial systems without any stenosis in epicardial coronary arteries. The patient's symptoms resolved almost completely with medical therapy. High volume shunts via coronary artery to left ventricular microfistulas may lead to increased volume overload and subsequent increase in end-diastolic pressure of the left ventricle and may cause left atrial enlargement.

## 1. Introduction

Coronary cameral fistulas (CCFs) are abnormal communications between a coronary artery and a heart chamber which are reported in less than 0.1% of patients undergoing diagnostic coronary angiography [1]. The patients are usually asymptomatic so these fistulas are detected incidentally during coronary angiography most of the time. However, if fistulas are widespread, they may cause exertional angina due to coronary steal phenomenon concomitant with left ventricular volume overload and left atrial enlargement. All three major coronary arteries are even less frequently involved in fistula formation.

## 2. Case Report

**Figure 1:**
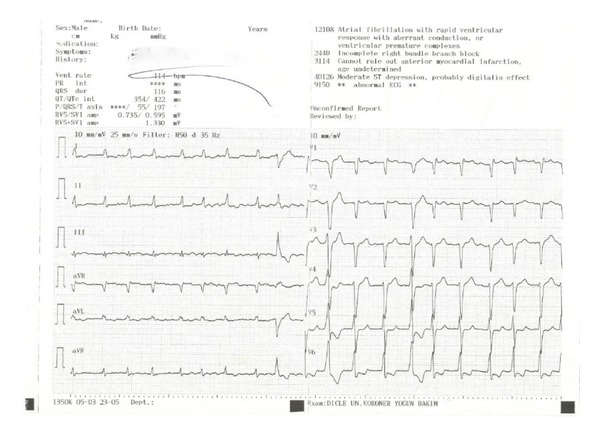
12-lead electrocardiogram of the patient on admission.

**Figure 2:**
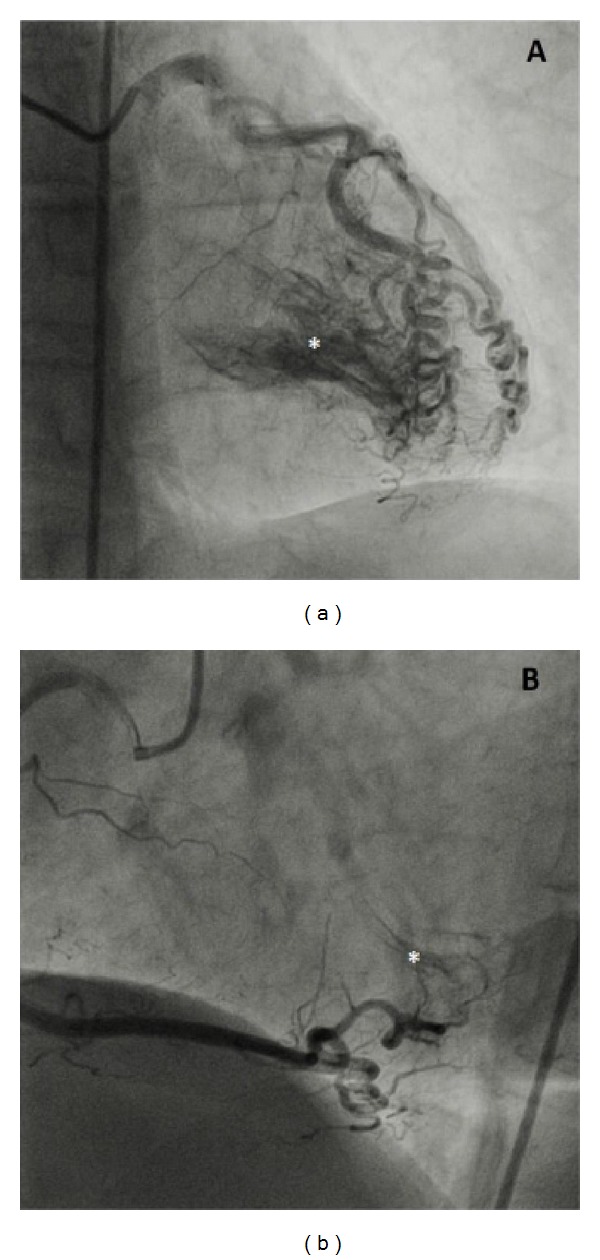
(a) Frames of anteroposterior oblique projection of left coronary angiogram depicting the CCFs opacifying the left ventricular cavity (asterisk) originating from the left coronary arterial system and (b) left oblique projection of right coronary angiogram demonstrating the opacification of the left ventricular cavity (asterisk) by microfistulae from the distal right coronary artery.

A 68-year-old woman was admitted to cardiology clinic with complaints of exertional dyspnea and angina for two years and a new onset palpitation. Physical examination was unremarkable except for irregular beats. Standard 12-lead electrocardiogram (ECG) revealed atrial fibrillation (AF) with a ventricular rate of 114 beat/minute and accompanying T wave abnormalities and minimal ST-depression on lateral derivations (Figure 1). The patient underwent transthoracic echocardiography (TTE) for the investigation of dyspnea and AF etiology. TTE examination was normal except for diastolic dysfunction and mild to moderate enlargement of left atrium (46 mm). Left atrial thrombus was ruled out with transesophageal echocardiography (TEE) and sinus rhythm was achieved by medical cardioversion with amiodarone infusion. Afterwards coronary angiography was performed which revealed diffuse and multiple coronary cameral fistulae originating from the distal segments of both left and right coronary arterial systems without any stenosis in epicardial coronary arteries (Video 1 and Video 2 in the Supplementary Material available online at http://dx.doi.org/10.1155/2014/475325) and significant amount of contrast media passing into left ventricular cavity through diffuse coronary cameral fistulae at each beat (Figure 2). Surgical or percutaneous closure of multiple and diffuse microfistulae is difficult technically. Also treatment of these fistulae is controversial [2]. A cardiovascular surgeon evaluated the patient and medical treatment was advised. Symptoms of our patient resolved almost completely under beta blocker therapy (50 mg of metoprolol daily). She was asymptomatic at the third month of followup.

## 3. Discussion

Coronary cameral fistulas (CCFs) are rare anomalies connecting coronary arteries to cardiac chambers or great vessels, which are detected rarely during routine angiographic evaluation. Frequency of congenital coronary fistulas is reported in 0.08% of 11350 adult patients undergoing diagnostic coronary angiography in Turkey [3] which is compatible with the literature (approximately 0.1%) [1]. The major sites of origin are the right coronary artery (55%), the left coronary artery system (35%), and both coronary arteries (5%). The main termination sites are the right ventricle (40%), right atrium (26%), and pulmonary arteries (17%). Less frequently they may drain into the superior vena cava or coronary sinus and least often into the left atrium or left ventricle [1, 4] as it is the case in our patient.

Although asymptomatic in the vast majority, CCFs may cause chronic myocardial ischemia and angina, congestive heart failure, myocardial infarction, pulmonary hypertension, rhythm disturbances, subacute bacterial endocarditis, thromboembolism, rarely rupture of aneurysmal segment, and sudden death [5]. Small fistulae usually do not cause any hemodynamic compromise. However, the larger and multiple fistulae may cause “coronary steal phenomenon” leading to myocardial ischemia [6]. The outcome with these connections depends upon the termination site. If termination is to the systemic venous side, it means a left-to-right shunt, and there will be left-sided volume overload when the termination is into left-sided cardiac structures. The volume of the shunt varies with the size of the fistula and the difference between systemic resistance and resistance in the terminating vessel/chamber. Blood flow moves from the coronary arteries to the lower pressure chambers/vessels. The best way to manage coronary cameral fistulae is not well known due to rarity of the condition [2]. Most patients are treated conservatively because symptoms relieve significantly with medical therapy especially beta blockers but not nitrates which may increase “coronary steal phenomenon” and worsen anginal symptoms in patients with CCFs [7].

To date there is only one case of CCF reported in association with paroxysmal AF [8]. In the present case without systemic hypertension, left atrial enlargement leading to AF may be due to increased volume overload passing through the coronary-cameral fistulae and subsequent increase in end-diastolic pressure of the left ventricle.

## Supplementary Material

Video 1. Antero-posterior oblique projection of left coronary angiogram depicting the CCFs opacifying the left ventricular cavity mimicking a left ventriculography.
Video 2. Left oblique projection of right coronary angiogram demonstrating contrast media leaking into left ventricle from the distal segment of right coronary artery through CCFs.
